# Tumor xenograft animal models for esophageal squamous cell carcinoma

**DOI:** 10.1186/s12929-018-0468-7

**Published:** 2018-08-29

**Authors:** Nikki P. Lee, Chung Man Chan, Lai Nar Tung, Hector K. Wang, Simon Law

**Affiliations:** Department of Surgery, The University of Hong Kong, Faculty of Medicine Building, 21 Sassoon Road, Pokfulam, Hong Kong

**Keywords:** Subcutaneous tumor xenograft, Orthotopic tumor xenograft, Patient-derived tumor xenograft, ESCC

## Abstract

Esophageal squamous cell carcinoma (ESCC) is the predominant subtype of esophageal cancer worldwide and highly prevalent in less developed regions. Management of ESCC is challenging and involves multimodal treatments. Patient prognosis is generally poor especially for those diagnosed in advanced disease stage. One factor contributing to this clinical dismal is the incomplete understanding of disease mechanism, for which this situation is further compounded by the presence of other limiting factors for disease diagnosis, patient prognosis and treatments. Tumor xenograft animal models including subcutaneous tumor xenograft model, orthotopic tumor xenograft model and patient-derived tumor xenograft model are vital tools for ESCC research. Establishment of tumor xenograft models involves the implantation of human ESCC cells/xenografts/tissues into immunodeficient animals, in which mice are most commonly used. Different tumor xenograft models have their own advantages and limitations, and these features serve as key factors to determine the use of these models at different stages of research. Apart from their routine use on basic research to understand disease mechanism of ESCC, tumor xenograft models are actively employed for undertaking preclinical drug screening project and biomedical imaging research.

## Background

### Esophageal squamous cell carcinoma (ESCC) and its management

Cancer is a life-threatening disease causing about 8 million deaths annually worldwide. Esophageal cancer ranks sixth on the list of top most common causes of cancer-related deaths, contributing approximately half a million deaths each year (GLOBOCAN 2012). Among different subtypes of esophageal cancer, ESCC is the predominant histological type and is highly prevalent in less developed areas especially certain regions in Asia and Africa. Multimodality treatment is offered to ESCC patients. Early stage patients with resectable tumors are treated with upfront tumor resection, while advanced patients on the other hand are first treated with chemotherapy/chemoradiation for tumor downstaging before surgical resection [1, 2]. Although this pre-surgery neoadjuvant treatment can achieve excellent response in subgroups of patients who are sensitive to chemotherapy/chemoradiation, about one-third of patients still exhibit only partial and suboptimal response. Even for responders of chemotherapy/chemoradiation, some of them may develop resistance in the later course of the treatment period. For those patients whose tumors are unresectable, and when the tumor is refractory to chemotherapy or radiotherapy, no effective treatment is available [3]. To provide more treatment options for patients not amendable by current therapies, new treatment approaches are proposed and evaluated in on-going clinical trials for their anti-tumor efficacies. Even with all these evolving and newly emerged treatment modalities, complete cure of disease is still difficult. In view of this, new treatments are urgently needed.

### The use of tumor xenograft animal models for preclinical research

The main purpose of developing tumor xenograft animal models for research is to bridge basic and clinical research and to supplement the use of in vitro model systems [4]. Tumor xenograft animal models provide a more sophisticated platform to study the process of tumorigenesis in an in vivo setting. This platform allows us to have a better understanding on the involvement of certain oncogenes or tumor suppressors in tumor development by uncovering their related signaling pathways and disease mechanisms [5]. Besides, the use of these models can provide us a research tool for preclinical drug response evaluation by determining the anti-tumor efficacies in addition to the drug toxicity, pharmacokinetics and pharmacodynamics [6]. Apart from drug response evaluation, these models can also facilitate biomedical imaging research by providing a model system for testing the usefulness and practicality on new tumor detection methods or reagents.

Mice are the most commonly used animals for tumor xenograft models because of several key advantageous features, such as the presence of comparable genome size with humans, short reproductive cycle, large litter size, low maintenance cost and ease of manipulation [5]. Different mouse strains in unique immunodeficiency backgrounds are used in cancer research, and these include athymic nude mice, SCID mice and NOD/SCID mice (SCID mice with an extra level of immunodeficiency). Among these strains, NOD/SCID mice demonstrate the best immunodeficiency due to the absence or defect in nearly all types of immune cells (B cells, T cells, dendritic cells, macrophages and natural killer cells), followed by SCID mice lacking B cells and T cells and then athymic nude mice without T cells [7]. Due to their various levels of immunodeficiency, different strains are considered for use for different research purposes. Taking into accounts the cost and features of different strains, athymic nude and SCID mice are preferably used for implanting human tumor cell lines, while SCID and NOD/SCID mice are rather used for the transplantation of human tumors.

### Commonly used tumor xenograft animal models for ESCC research

**Fig. 1:**
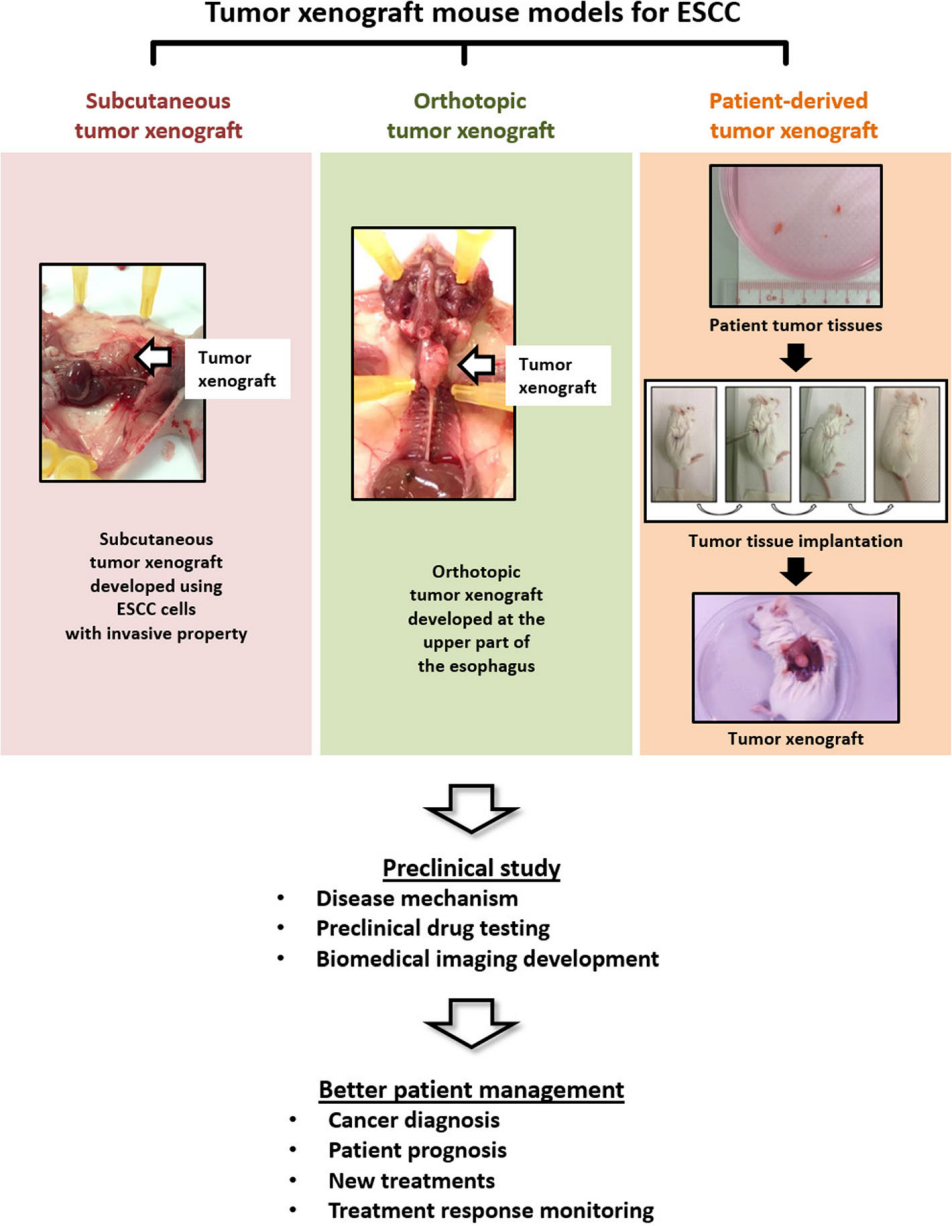
The preclinical use of tumor xenograft mouse models for ESCC research. Different types of tumor xenograft mouse models are developed for ESCC research, which include subcutaneous tumor xenograft model, orthotopic tumor xenograft model and patient-derived tumor xenograft model (the diagram shows the development of this model in a subcutaneous way). These models are commonly used in preclinical research to understand disease mechanism, to perform preclinical drug testing and to develop biomedical imaging. Results derived from the use of these models can advance cancer research and can lead to better patient management in the aspects of cancer diagnosis, patient prognosis, new treatment development and development of new treatment response monitoring methods. The use of animals for research has been approved by the Committee on the Use of Live Animals in Teaching and Research (CULATR) of our institute.

Three types of tumor xenograft animal models for ESCC research are developed by implanting ESCC cells/xenografts or patient tumors in immunodeficient animals, namely subcutaneous, orthotopic and patient-derived tumor xenograft model (Fig. 1). Each of them has its own strengths and weaknesses in terms of model features (Table 1), establishment methods (Table 2) and preclinical utilities (Table 3), which place them in a unique position for early stage, mid stage or late stage research [8, 9].

**Table 1:** Key features of tumor xenograft models for ESCC research

Tumor xenograft models	Key features
Subcutaneous	*Advantages*
	• easy to establish • low cost • palpable tumor for easy tumor size measurement
	*Limitation*
	• incorrect tumor microenvironment
Orthotopic	*Advantage*
	• correct tumor microenvironment
	*Limitations*
	• technique demanding • high cost • specialized imaging systems required for tumor size measurement
Patient-derived	*Advantage*
	• high resemblance to patient tumor
	*Limitations*
	• long latency period • limited engraftment rate • high cost • use of patient specimen

This table lists the key features of different tumor xenograft models for ESCC research based on comprehensive reviews on tumor xenograft models for cancer research [8, 35]

**Table 2:** Technical details for establishing ESCC tumor xenograft models

Tumor xenograft model	Technical details	References
Subcutaneous	Starting material: human ESCC cell line	[36, 37]
	Injected cell number: 2 × 10^6^	
	Mouse strain: nude, SCID, NOD/SCID	
	Cell injection site: flank	
Orthotopic	Starting material: Human ESCC cell line	[23]
	Mouse strain: nude	
	Tumor development site: cervical and abdominal esophagus	
Patient-derived	Starting material: Human ESCC tumor tissue	[37, 38]
	Mouse strain: NOD/SCID	
	Tumor implantation method: subcutaneous	

This table shows some examples from representative publications and does not mean to be inclusive

**Table 3:** Uses of different tumor xenograft models for preclinical drug testing for ESCC

Tumor xenograft models	Test compounds/drugs	References
Subcutaneous	Temsirolimus	[14]
Subcutaneous	YQ23 alone or combined with cisplatin or 5-fluorouracil	[16]
Subcutaneous	Ginsenoside Rg3 alone or combined with paclitaxel and cisplatin	[15]
Subcutaneous	Afatinib	[39]
Orthotopic	Temsirolimus	[14]
Patient-derived	Lapatinib alone and in combination with 5-fluorouracil or oxaliplatin	[25]
Patient-derived	Cisplatin and 5-fluorouracil	[26]
Patient-derived	Trastuzumab	[27]

#### Subcutaneous tumor xenograft model

Subcutaneous tumor xenograft model is a classical animal model for ESCC research. This model is established by implanting ESCC cells/xenografts under the skin of the immunodeficient animals to develop subcutaneous tumors. The procedure of establishing subcutaneous tumor is technically simple as it only involves needle injection of ESCC cells or direct implantation of ESCC xenografts under the skin of animals. The growth of subcutaneous tumors can be performed non-invasively by using an electronic caliper to measure the palpable tumors. These technical procedures of tumor establishment and monitoring can maintain the reproducibility and effectiveness (both time and cost) of this model. Despite these advantages, this model suffers certain limitations as it does not fully represent the clinical situation. For instance, this model associates reduced tumor heterogeneity as in most scenarios homogeneous ESCC cell lines are used as the source material. Besides, subcutaneous tumors do not grow in their native tumor microenvironment and this makes them not suitable for the study of tumor-stromal interactions. With such strengths and weaknesses [8], this model is mainly used in early stage research to study the biology and mechanism of ESCC tumorigenesis.

Accumulating reports have revealed the mileage of applying subcutaneous tumor xenograft model for early stage research by using it to study tumor properties of ESCC-related molecules and their associated disease mechanisms. Overexpression of microRNA-340, a microRNA downregulated in ESCC tumors, in EC9706 ESCC cells inhibited the growth properties of these cells in a subcutaneous tumor xenograft model. The effect of this microRNA on ESCC was mediated in part via its effect on a protein transferase PSAT1, which was identified in the same study as a direct target of microRNA-340 [10]. Another study demonstrated faster growth of subcutaneous tumors derived from KYSE-30 ESCC cells with overexpression of a matrix metalloproteinase MMP1 when compared to the control cells. In addition, these MMP1-overexpressing ESCC cells also exhibited metastatic potentials. The ability of MMP1 to promote tumor progression and metastasis was concordantly revealed to be due to its stimulating effect on a tumorigenic pathway involving PI3K and AKT [11]. A separate report also utilized subcutaneous tumor xenograft model to reveal cellular retinoic acid binding protein 2 (CRABP2) as a tumor suppressor by showing slower growth rate of subcutaneous tumors derived from EC109 ESCC cells overexpressing CRABP2 when compared to the control experimental group [12]. These studies collectively signify the usefulness of this model on studying ESCC-related molecules and their related mechanisms in ESCC.

Apart from the use of subcutaneous tumor xenograft model for tumor biology study, attempts have been made for using this model to investigate the anti-tumor efficacies of new treatment methods or compounds/drugs for treating ESCC. Subcutaneous tumor-bearing mice were subjected to treatment to induce the expression of an ESCC prognostic marker microRNA-375 and smaller tumor size was obtained at the end of the experiment when compared to control experiment [13]. Apart from the routine use to examine new treatment methods, this model was also applied to investigate the anti-tumor efficacies of new compounds/drugs for counteracting ESCC, such as temsirolimus currently used for renal cell carcinoma patients [14]. Besides its utility on anti-tumorigenecity testing, this model can be applied to examine the chemo-sensitizing effects of test compounds. Treatment of subcutaneous tumor-bearing animals with Ginsenoside Rg3, an ingredient extracted from ginseng, provided evidence demonstrating the sensitizing effects of this compound to two chemotherapeutic drugs paclitaxel and cisplatin commonly used for ESCC patients [15]. More recently, a novel oxygen carrier YQ23 was shown to exert chemo-sensitizing effects selectively on chemo-resistant subcutaneous SLMT-1 ESCC xenografts, but not chemo-sensitive HKESC-2 ESCC xenografts, in treatment plans using cisplatin or 5-fluorouracil, which are also traditional chemotherapeutic drugs used for ESCC patients [16]. The above studies examining the anti-tumor efficacies of test compounds/drugs alone or in combination with common chemotherapeutic drugs used for ESCC have highlighted the preclinical utility of subcutaneous tumor xenograft model for compound/drug testing.

Taken together, subcutaneous tumor xenograft models particularly those well characterized for their sensitivities towards chemotherapeutics currently used for ESCC are valuable research tools for studying tumor biology and disease mechanisms of ESCC in an in vivo setting and for performing preclinical anti-tumor efficacy studies (Table 3).

#### Orthotopic tumor xenograft model

Orthotopic tumor xenograft model is an alternate animal cancer model used for ESCC research. This model is established by implanting ESCC cells/xenografts in the esophagus of the immunodeficient animals to develop orthotopic tumors. The procedure of establishing orthotopic tumors is more technically demanding when compared to those needed for establishing subcutaneous tumors, as it requires small animal surgery and/or anesthesia. Two main approaches are employed to implant ESCC cells/xenografts in the animal esophagus, for which they differ at the site of tumor implantation. One method implants ESCC cells/xenografts in the upper region of the esophagus [17, 18], while the other implants ESCC cells/xenografts in the lower end of the esophagus at the abdominal region near the gastroesophageal junction [19–22]. Recently, we have also reported the survival comparison between animals with orthotopic tumor developed at the upper esophageal region and the lower region, and found that those with tumor at the abdominal region have better survival [23]. Although it is more time and labor intensive to establish orthotopic tumors, this model can accommodate the study of tumor growth in a correct tumor microenvironment as in the clinical situation and enable the study of tumor-stromal interactions [8]. However, it also suffers from the same limitation as the subcutaneous model by using ESCC cell lines as the source materials for tumor establishment. Another major disadvantage of this model is the need to use specialized imaging methods, e.g. the in vivo imaging system coupled with the use of bioluminescent technology [19, 20, 22], to monitor tumor growth. Despite these limitations, the strengths of this model make it a valuable animal model for use in mid to late stage research.

Although orthotopic tumor xenograft model offers superior advantages in comparing with the subcutaneous model, its use remains restricted due to the above mentioned limitations. Despite these shortcomings, an expanding number of reports have exemplified the application of this model for studying ESCC tumorigenesis and for performing preclinical anti-tumorigenecity testing. When this model was used to reveal the tumor suppressing effect of a transmembrane protease DESC1, slower tumor growth rate was observed with the use of DESC1-expressing KYSE-150 ESCC cells when compared to those using control cells [22]. In an independent study using this model to examine the tumor phenotype of a tumor-related protein kinase AKT, an obvious tumor-suppressing effect was found associated with the knockdown of AKT, such that a reduced growth rate of orthotopic tumors was readily detected [19]. Aside its use to study tumor growth, this model can be applied to investigate the mechanism of tumor invasion. A comprehensive study pointed out the involvement of a cell surface molecule CD44H on tumor invasion based on the invasive patterns observed between orthotopic tumors derived from T.T ESCC cells and the invasive counterpart T.T-1 ESCC cells. T.T-1 ESCC cell line expressed high level of CD44H and was derived from tumor cells isolated from cervical lymph node in a T.T ESCC cells-derived orthotopic tumor xenograft model [17]. On top of its use to study tumor growth and invasion, this model is capable to assess the effects of test compounds on post-treatment survivals. A prolonged survival was observed in orthotopic ESCC tumor-bearing animals after treatment with an mTOR inhibitor temsirolimus, for which this inhibitor is currently used for renal cell carcinoma [14].

Generally, orthotopic tumor xenograft model supplements the regular use of subcutaneous model for the study of ESCC by providing a correct tumor microenvironment. In addition, this model functions as an indispensable tool in preclinical research for examining the anti-tumor effects of test compounds/drugs (Table 3).

#### Patient-derived tumor xenograft model

Patient-derived tumor xenograft model is a more advanced animal cancer model than the above mentioned models for ESCC research. This model is established with the use of ESCC tumors resected from patients to develop tumor xenografts in immunodeficient animals. The patient tumors can be implanted subcutaneously or orthotopically. Depending on the tumor implantation site, this model also retains certain features as the subcutaneous and orthotopic tumor xenograft models. Remarkably, tumor xenografts derived from patient tumors have preserved genetic, histological and phenotypic properties as the donor tumors. Since the stromal and cell components of the patient-derived tumor xenografts are maintained as in the donor tumors, the use of this model can exclude the disadvantages associated with the use of homogeneous tumor cell lines. Despite these advantages, this model inevitably suffers from several limitations [5, 7, 8]. The model establishment requires the use of resected patient tumors, for which some basic research laboratories may not have access to this specimen source. Besides, the engraftment rates of the patient tumors to form tumor xenografts are suboptimal and vary depending on a number of factors, such as tumor types, tumor implantation sites, mouse strains, tumor features and patient features [7]. Even for a patient tumor that can be successfully engrafted, it requires a long latency period to grow into a tumor xenograft and sometimes this process can last for as long as 6 months. Therefore, the above limitations have made the tumor establishment process a high cost and labor intensive procedure. Despite these downsides, this model is gradually replacing other animal cancer models to be used in mid and late stage research and is especially beneficial for the preclinical evaluation of anti-tumor efficacies of new compounds/drugs. Indeed, this model is sometimes referred to “clinical trials in a mouse” due to its superior power to predict clinical response of test compounds/drugs due to the high resemblance between tumor xenografts and donor tumors [8].

Considering all the pros and cons, patient-derived tumor xenograft model can provide us a research platform not only for studying the disease mechanisms of ESCC but can also facilitate preclinical drug screening. For the latter application, this model is more preferably used than the subcutaneous and orthotopic models due to its high clinical relevance, which supports its use on evaluating the anti-tumor efficacies of new compounds/drugs. In this area, individual research teams have established their own collections of patient-derived tumor xenograft models with preserved tumor heterogeneity for screening drugs or drug combinations for ESCC [24–27] (Table 4).

**Table 4:** Collections of patient-derived tumor xenograft models for ESCC

Number of established models	Characterized deregulations	References
37	EGFR, K-ras, B-raf and PIK3CA mutation HER2 expression	[26]
25	HER2 expression and amplification	[27]
5	EGFR, K-ras, B-raf and PIK3CA mutation HER2 expression and amplification	[27]

Abbreviations used: *EGFR* epidermal growth factor receptor, *HER2* human epidermal growth factor receptor 2 *PIK3CA* phosphatidylinositol-4,5-bisphosphate 3-kinase catalytic subunit alpha

The patient-derived tumor xenograft models with well characterized molecular deregulations commonly found in ESCC can be used as a tool for testing currently used drugs for their new uses on ESCC. This process is important as it can research for new drugs for patients who are resistant to current drug treatments. Cisplatin and 5-fluorouracil are two chemotherapeutic drugs used for ESCC, however not all patients have good drug responses. To have a better understanding on the drug mechanism, Zhang et al. established a panel of patient-derived tumor xenograft models and well characterized them for common genetic aberrations frequently detected in ESCC, such as HER2 expression and mutations of EGFR (epidermal growth factor receptor), K-ras, B-raf and PIK3CA (phosphatidylinositol-4,5-bisphosphate 3-kinase catalytic subunit alpha). Using a panel of xenografts with well characterized HER2 and PIK3CA status to examine the treatment effect of cisplatin and 5-fluorouracil, tumor xenografts negative for HER2 and carrying wild-type PIK3CA were more sensitive to such treatment when comparing to HER2-positive xenografts irrespective of the mutation status of PIK3CA [26]. Results derived from this study have revealed the link between tumor genetic compositions and chemotherapeutic drug responses.

Apart from the use of patient-derived tumor xenograft models with defined genetic compositions for testing conventional chemotherapeutic drugs, these models have been used to examine the anti-tumor efficacies of drugs that are not clinically used for ESCC, such as trastuzumab and lapatinib. Testing the effect of trastuzumab on patient-derived tumor xenografts revealed HER2-positive ESCC was responsive to such treatment, but not for those carrying concurrent PIK3CA mutation. Further treatment of these HER2-positive and PIK3CA-mutated tumor xenografts with AKT inhibitor AZD5363 subsequently rendered the xenografts to be responsive to trastuzumab treatment again [27]. Another study examined the sensitizing effect of lapatinib on chemotherapeutic drugs oxaliplatin or 5-fluorouracil using a patient-derived tumor xenograft model. Combined treatment of lapatinib with 5-fluorouracil led to a more potent growth inhibitory effect than lapatinib alone or its combined treatment with oxaliplatin [25]. These studies have clearly presented the usefulness of these models for preclinical drug testing. Importantly, such testing on tumor xenografts with defined genetic backgrounds can facilitate the development of precision medicine by selecting drug treatment based on the genetic deregulations of the tumors.

Patient-derived tumor xenograft models can mimic the genetic diversity and composition of the clinical settings due to the high histological and pathological relevance between donor tumors and the established tumor xenografts. These earlier studies have put forth the preclinical application of these models for evaluating the anti-tumor efficacies of different drugs/compounds (Table 3). Derived results can also provide solid evidences supporting the use of new drugs/compounds for treatment of ESCC. Such preclinical test therefore forms a vital platform prior to clinical trials.

## Conclusions

### Conclusions and future perspectives

Tumor xenograft animal models remain indispensable tools for biomedical research and provide a fundamental platform for preclinical drug screening. Mainly, three broad types of tumor xenograft models, i.e. subcutaneous, orthotopic and patient-derived, are available and routinely used for ESCC research. Although these models are established in immunodeficient animals using human ESCC cells/xenografts/tissues, each of them indeed associate distinct advantages and disadvantages. The unique features of each model support its respective use in different stages of research. However, care must be taken when analyzing the results derived from different tumor xenograft studies as certain variables can affect the result interpretation and reproducibility. These variables can be tumor implantation sites, tumor properties, tumor origins and several others [6]. To expedite the utility of tumor xenograft models for preclinical drug testing, extra efforts should be dedicated to define fully the treatment sensitivities of ESCC cells/xenografts/tissues used for establishing tumor xenografts, such that the established xenografts can be used to represent specific clinical conditions. In addition, such characterized models can also provide a working platform to examine the treatment-sensitizing effects of test compounds/drugs.

Aside the above applications, tumor xenograft models can also be used to address new challenges on biomedical imaging. New imaging modalities such as optical coherence tomography can be examined and validated in various tumor xenograft models for their utility to detect tumors. Specifically for optical coherence tomography, the incorporation of contrast agent can enhance the detection capability on tumors and even for detecting early stage tumors [28]. Active research is devoted in this area for ESCC research. Initial attempt has been made to deploy the capacity of optical coherence tomography to reveal various layers of the mouse esophageal wall in a high resolution manner [29]. Such effort has launched the research towards this direction by further examining the image contrast-enhancing effects associated with contrast agents like nanoparticles [30]. In all, tumor xenograft models provide an ideal and versatile platform for ESCC research and can facilitate the research on tumor biology, preclinical drug testing and possibly biomedical imaging.

Although tumor xenograft models developed in immunodeficient mice are widely used in cancer research due to their low cost, the use of this animal type is suffered from the lack of immunity. Therefore, they have limited use in research that requires an intact immune system. Alternatively, this limitation can be addressed with the use of humanized mice, for which these mice are generated with a human immune system [31, 32]. The use of humanized mice can further enhance the clinical relevance of the patient-derived tumor xenograft model and is particularly useful to conduct immunotherapy study [31, 33, 34]. Another type of animal model that is gaining wide spread use is genetically engineered mice model that are generated by transgenic technologies [31, 33]. Together, these two additional types of animal models can be utilized in parallel to the tumor xenograft models to further enhance result interpretation and clinical relevance of the study.

## Abbreviations

CRABP2: Cellular retinoic acid binding protein 2
EGFR: Epidermal growth factor receptor
ESCC: Esophageal squamous cell carcinoma
PIK3CA: Phosphatidylinositol-4,5-bisphosphate 3-kinase catalytic subunit alpha
